# Discrimination of Closely Related *Vibrio* Strains by Label-Free Surface-Enhanced Raman Spectroscopy (SERS)

**DOI:** 10.3390/biology15151300

**Published:** 2026-08-05

**Authors:** Aini Nadia Mazlan, Nathaniel Leong, Annie Christianus, Zuraidah Zan, Norazrin Ariffin, Junfeng Xie, Annita Seok Kian Yong, Chou Min Chong

**Affiliations:** 1 Institute of Bioscience, Universiti Putra Malaysia, Serdang 43400, Selangor, Malaysia; gs66922@student.upm.edu.my; 2 Wireless and Photonics Networks, Research Centre, Faculty of Engineering, Universiti Putra Malaysia, Serdang 43400, Selangor, Malaysia; gs71203@student.upm.edu.my; 3 Department of Aquaculture, Faculty of Agriculture, Universiti Putra Malaysia, Serdang 43400, Selangor, Malaysia; annie@upm.edu.my; 4 Department of Computer and Communication Systems, Faculty of Engineering, Universiti Putra Malaysia, Serdang 43400, Selangor, Malaysia; zuraidahz@upm.edu.my; 5 Department of Agriculture Technology, Faculty of Agriculture, Universiti Putra Malaysia, Serdang 43400, Selangor, Malaysia; norazrin@upm.edu.my; 6 State Key Laboratory of Biocontrol, China-ASEAN Belt and Road Joint Laboratory on Mariculture Technology, School of Life Sciences, Sun Yat-sen University, Guangzhou 510275, China; xiejf@mail.sysu.edu.cn; 7 Institut Marin Borneo, Universiti Malaysia Sabah, Jalan UMS, Kota Kinabalu 88400, Sabah, Malaysia; annitay@ums.edu.my; 8 Laboratory of Sustainable Aquaculture (AquaLab), International Institute of Aquaculture and Aquatic Sciences (I-AQUAS), Universiti Putra Malaysia, Port Dickson 71050, Negeri Sembilan, Malaysia

**Keywords:** Surface-Enhanced Raman Spectroscopy (SERS), *Vibrio alginolyticus*, *Vibrio parahaemolyticus*, *Vibrio vulnificus*, principal component analysis (PCA), linear discriminant analysis (LDA), partial least squares discriminant analysis (PLS-DA)

## Abstract

Fish pathogens pose significant challenges to aquaculture, causing high mortality and economic losses. Thus, rapid, accurate, and cost-effective identification methods for disease management are needed. Conventional bacterial identification techniques are time-consuming and costly. Raman spectroscopy is an emerging alternative, enabling species differentiation based on molecular signatures. This study investigates surface-enhanced Raman spectroscopy (SERS) combined with principal component analysis with linear discriminant analysis (PCA-LDA) and partial least-squares discriminant analysis (PLS-DA) to classify three relevant *Vibrio* species: *Vibrio alginolyticus*, *Vibrio parahaemolyticus*, and *Vibrio vulnificus*. SERS spectra from bacterial cultures were analyzed to identify species-specific variations. Distinct clustering was observed, with *V. vulnificus* showing stronger amide-related signals, *V. parahaemolyticus* exhibiting higher sulfur-related Raman peaks, and *V. alginolyticus* showing distinct lipid region signals. The PCA-LDA model achieved 100% classification and 97.06% cross-validation accuracy under standardized laboratory conditions in this controlled proof-of-concept study using spectral replicates obtained from cultured laboratory bacterial stocks. The PLS-DA model showed excellent discriminatory performance (AUC = 1.00), with strong calibration for two species (R^2^ > 0.80) and slightly lower for *V. alginolyticus* (R^2^ = 0.71). These findings demonstrate that SERS combined with multivariate analysis is a promising approach for rapid and label-free *Vibrio* identification and classification under controlled laboratory conditions.

## Simple Summary

Bacterial diseases are among the major challenges in sustainable aquaculture production, which can spread rapidly, as well as cause fish mortality and major losses in fish production. A problem in current diagnostic methods is slow, expensive, and sometimes unable to clearly differentiate between closely related bacterial species, which delays in disease control and prevention. Therefore, this study aimed to develop a faster, low-cost, and more reliable method for identifying fish pathogens commonly found in marine aquaculture systems. A laser-based analytical technique, surface-enhanced Raman spectroscopy, was used to obtain distinct biochemical fingerprints from the bacterial cells, while computational pattern-recognition methods were used to analyze, compare, and classify the resulting based on the spectral signals. The high classification performance was demonstrated using cultured bacterial samples under controlled laboratory conditions, suggesting the potential of this approach as a proof-of-concept method for bacterial identification. Overall, this study demonstrates the potential of this combined approach as a rapid and accurate method for bacterial classification under controlled laboratory conditions and supports its future development for aquaculture disease management.

## 1. Introduction

Aquaculture has emerged as one of the fast-growing and most reliable sectors in global seafood production. This industry plays a crucial role in addressing the increasing global demand for protein-rich food. However, the sustainability and profitability of aquaculture are significantly threatened by the frequent occurrence of disease outbreaks, which lead to substantial economic losses for farmers and negative impacts on national economies [1,2].

Among the various pathogens affecting aquaculture, bacterial infections, particularly those caused by *Vibrio* species, pose a serious threat to fish health and farm production. Consequently, there is a need for a rapid and accurate detection tools that can identify pathogenic agents at an early stage, thereby accelerating early intervention and disease management [2,3].

Traditionally, detection and identification of bacterial pathogens in aquaculture systems rely on classical microbiological methods, such as selective culture media, Gram staining, and biochemical assays [4,5]. These methods were widely used but often fall short in accurately distinguishing between bacterial species due to the biochemical and physical similarities shared by those closely related organisms [6]. For example, thiosulphate citrate bile salts sucrose (TCBS) agar is commonly used to isolate *Vibrio* species; however, it is unable to distinguish closely related species such as *Vibrio alginolyticus*, *Vibrio parahaemolyticus*, and *Vibrio vulnificus*. Studies have shown that TCBS agar can support the growth of distantly related bacterial groups, leading to false positives [6,7,8]. These limitations in specificity can delay accurate diagnosis and allow infections to progress, resulting in increased fish mortality and economic loss.

Molecular techniques such as PCR, microarrays, and RNA sequencing have been introduced to overcome those problems by offering improved sensitivity and specificity [9,10]. However, their effectiveness largely depends on the selection of a specific target gene. They are also usually used for targeting only certain bacteria species that are widely distributed and stable within the genome. Moreover, the selection of tissue for sampling can significantly affect diagnostic outcomes [11]. Fish tissues often contain inhibitory substances such as hemoglobin, bacterial components, and high levels of non-target DNA, all of which can interfere with amplification and hinder the reliability of molecular diagnostics [12,13]. These molecular methods are also time-consuming, labor-intensive, and costly, further limiting their routine application in farm-level disease observation [12].

Based on these challenges, alternative diagnostic approaches have been investigated. One alternative technique is Surface-Enhanced Raman Spectroscopy (SERS), a powerful vibrational spectroscopic method that allows for rapid, sensitive, and label-free identification of molecular structures [14]. Raman spectroscopy operates by detecting the inelastic scattering of photons when the monochromatic laser light interacts with molecular bonds in the sample [15]. When photons are absorbed, transmitted, or scattered by the sample, distinct vibrational signatures are produced based on the specific molecular composition [16,17].

SERS enhances these signals by using the interaction between light and noble metal nanostructures, such as gold, silver, or copper, dramatically increasing the sensitivity of Raman spectroscopy to the level of single-molecule detection [18,19]. In SERS, gold or silver nanoparticles (NPs) is commonly used to fabricate the substrates, and each NP shape produces electromagnetic “hot spots” that amplify signals differently [20]. These enhanced signals are highly specific to molecular vibrations associated with chemical bonds and functional groups, such as C-H, N-H, and O-H, enabling the detection of complex biomolecules, including proteins, nucleic acids, lipids, and carbohydrates [21,22]. The region between 400 and 1800 cm^−1^, often referred to as the molecular fingerprint regions, is particularly informative in this context [22,23].

SERS offers several advantages over traditional and molecular methods, including high sensitivity, molecular specificity, rapid processing time, and non-destructive analysis [24]. These benefits make it an attractive tool for studying complex biological systems, including those relevant to aquaculture [24,25]. Raman spectroscopy has previously demonstrated its ability in profiling a range of prokaryotic and eukaryotic cells, showcasing its potential for bacterial identification in clinical and environmental area [26,27,28,29,30,31].

However, despite it, it is important to note that Raman spectroscopy, including SERS, typically differentiates at the genus level and may not always be capable in distinguishing between closely related bacterial species due to their very similar biochemical compositions, which produce almost identical Raman spectral signals. Yet, when combined with advanced data-analysis techniques such as principal component analysis (PCA) and linear discriminant analysis (LDA), the discriminatory power of SERS can be significantly improved. It is because multivariate methods such as principal component analysis (PCA) reduce high-dimensional SERS spectral data into orthogonal components that capture the greatest variance. Meanwhile, linear discriminant analysis (LDA) maximizes between-class variance relative to within-class variance. Therefore, by combining this method, it is possible to enhance the subtle biochemical differences that are masked by spectral overlap, noise, and experimental variability in raw SERS data [32].

In addition, partial least-squares discriminant analysis (PLS-DA) has emerged as a complementary supervised learning method that not only enables classification but also provides predictive calibration models and validation through tools such as Receiver Operating Characteristic (ROC) curves [33]. Previous studies have highlighted the strength of combining PLS-DA with vibrational spectroscopy for robust bacterial identification and quantification [34,35,36].

A label-free colloidal SERS technique, coupled with PCA-LDA and PLS-DA, was employed to differentiate and classify three *Vibrio* strains (*V. alginolyticus*, *V. parahaemolyticus*, and *V. vulnificus*) isolated from aquaculture farms. A significant result of this study is that the label-free colloidal SERS combined with PCA-LDA and PLS-DA was able to discriminate and classify of three closely related *Vibrio* species and demonstrating high sensitivity and specificity. This integrated chemometric approach offers a rapid and reliable solution for profiling bacterial samples, overcoming the limitations of traditional omics tools and classical immunoassays that often require hard work, time consuming, and economically burdensome.

## 2. Materials and Methods

### 2.1. Preparation of Bacterial Strains

Three bacterial species were used in this study, including *Vibrio* alginolyticus, *Vibrio parahaemolyticus*, and *Vibrio vulnificus*. These Gram-negative bacteria were obtained from the Laboratory of Fish Health and Disease, Department of Aquaculture, Universiti Putra Malaysia. All bacteria had been harvested from infected fish and previously identified using 16S rRNA sequencing. The bacterial strains from the −80 °C glycerol stock were isolated on a nutrient agar plate for approximately 12 h prior to the experiments to revive the bacteria. Subsequently, the bacteria were inoculated cultured on thiosulfate citrate bile salt agar (TCBS) (Merck KGaA, Darmstadt, Germany), a selective agar for *Vibrio* species. The TCBS agar was prepared according to the instruction on the bottle label. The *Vibrio* bacterial cultures on the TCBS agar were incubated at 30 °C for 24 h. The growth morphology of three *Vibrio* strains on TCBS agar plates can be observed on Figure S1. A single colony was then inoculated into 10 mL of fresh broth media (tryptic soy broth (TSB) supplemented with 2% NaCl) (Becton, Dickinson and Company, Franklin Lakes, NJ, USA) and incubated at 30 °C for 18 h with continuous shaking at 200 rpm. The optical density (OD) was measured after the 18 h of incubation. To estimate the number of viable bacteria, colony-forming units per milliliter (CFUs/mL) were determined using the 10-fold serial dilution plate method, as described by [37]. After incubation, colonies formed on the plates were counted, and only plates with 30 to 300 colonies were considered for accuracy determination. The concentration of bacteria in CFU/mL was calculated using the following formula: CFU/mL = (Number of Colonies Counted)/(Volume plated in the agar × Dilution Factor). The estimated CFUs/mL and the OD were recorded for bacteria sample preparation. All steps were performed separately for each bacterium until the estimated concentration of each bacterium was achieved.

### 2.2. Sample Preparation

**Figure 1:**
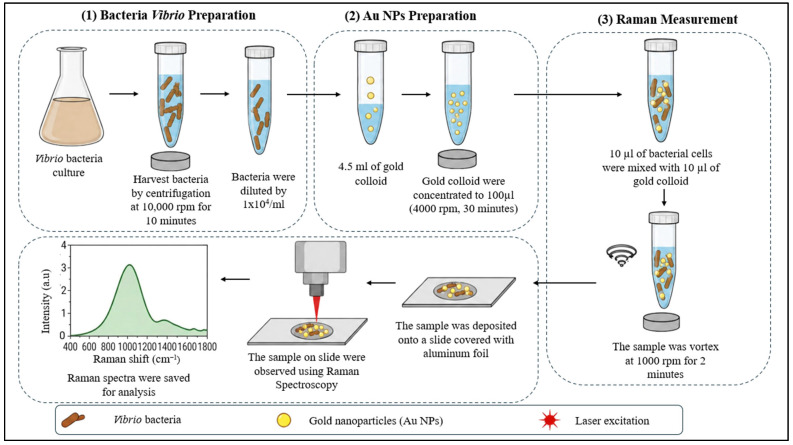
Workflow of sample preparation for acquisition of surface-enhanced Raman spectroscopy (SERS) spectra from *Vibrio* bacteria at 1 × 10^4^ CFU/mL.

The bacterial cells that incubated in broth media for 18 h were harvested by centrifugation at 10,000 rpm for 10 min using an Eppendorf 5810R refrigerated centrifuge (Eppendorf SE, Hamburg, Germany), then washed and resuspended in phosphate-buffered saline (PBS) (Sigma-Aldrich, St. Louis, MO, USA). The bacterial cells were washed three times before being used for Raman measurements. Before being used, bacterial cells were diluted to an approximate concentration of 1 × 10^4^ cells/mL with PBS for each sample. Sample preparation for Raman measurement was carried out based on the protocol by [38], with some modifications. An overview of the sequential sample preparation and mixing steps is illustrated in Figure 1. The gold nanoparticles, 40 nm in diameter (Sigma-Aldrich, St. Louis, MO, USA), underwent a washing process by centrifugation (4000 rpm, 30 min) using an Eppendorf 5810R refrigerated centrifuge (Eppendorf SE, Hamburg, Germany) and were redispersed in deionized water. This process was repeated three times. For the SERS analysis, 4.5 mL of the gold colloid that were washed previously were concentrated to 100 µL through centrifugation (4000 rpm, 30 min), and the requisite amount of supernatant was removed, after which the gold was ready to be used. Subsequently, 10 µL of gold nanoparticles were mixed with 10 µL of *Vibrio* bacterial cells. The resulting solution was vortexed (1000 rpm) using VELP Scientifica ZX3 Advanced Vortex Mixer (VELP Scientifica Srl, Usmate Velate, Italy) for 2 min and incubated for an additional minute before being deposited onto a glass slide that was covered with aluminium foil for SERS analysis [39]. The sample solution placed on the slide was in a dome-shaped droplet to ensure the raman probe touches the sample instead of the slide surface. The vortexing step introduced forced convection to facilitate a more uniform formation of aggregates, rather than relying on the random Brownian motion of the molecules for aggregate formation [40].

### 2.3. SERS Spectral Acquisition and Preprocessing

The vortexed mixture deposited for measurement was incubated for 5 min on a microscope glass slide covered with aluminium foil prior to spectral acquisition [39]. A micro-Raman spectrometer equipped with a confocal microscope and a 785 nm narrow linewidth laser source (XIM-6206-785-500-2, Yixi Intelligent Technology, Hangzhou, China) with an output power of 75 mW at the fibre ferrule was used, with the integration time maintained at 10 s. The spectra for each sample were recorded after the 10 s integration time. To improve spectral quality, the acquired spectra were smoothed using the Savitzky-Golay algorithm with a 13-point window and a third-order polynomial, effectively reducing shot noise while preserving key spectral features [41].

The pre-processing of the SERS spectra using OriginLab Pro version 2024 (OriginLab Corporation, Northampton, MA, USA) software involved multiple steps. Initially, the Daubechies wavelet of order 10 was applied as the mother wavelet with a thresholding level of 50%. Baseline correction was carried out using the asymmetric least-squares smoothing algorithm (asymmetric factor: 0.001, threshold: 0.01, smoothing factor: 2, number of iterations: 10), followed by subtraction of a constant value corresponding to the minimum of each spectrum to set the baseline to zero. The spectra were then normalized to the laser emission peak at 0 cm^−1^, and the spectra were cropped to the fingerprint region, which is 400–1800 cm^−1^. The spectral region near 400 cm^−1^ was visually inspected after preprocessing to confirm that no baseline-correction artefacts or abnormal signals were present. The spectra before and after cropping were compared to confirm that removal of the edge regions did not affect the spectral profile or important Raman bands. After the spectra cropped, the baseline correction was repeated using the same parameters to ensure uniform zero baselines across all spectra within the fingerprint region. Additional to the pre-processing, spectral area normalization and first derivative transformation was performed. The preprocessing steps were applied prior to multivariate analysis.

Subsequently, the normalization data were transformed into Z-score, and principal component analysis (PCA) was conducted using OriginLab Pro version 2024 (OriginLab Corporation, Northampton, MA, USA) to reduce the dimensionality of the SERS data. PCA scores of the first and second principal components were utilized to construct two-dimensional plots and linear discriminant analysis (LDA) for evaluating the degree of similarity or variation among the SERS spectra of different *Vibrio* strains. The accuracy of the PCA-LDA model was then tested using leave-one-out cross-validation.

### 2.4. PCA-LDA Model Development and Classification Performance

The preprocessed SERS spectra were transformed into Z-score, and principal component analysis (PCA) was conducted using OriginLab Pro version 2024 (OriginLab Corporation, Northampton, MA, USA) to reduce the dimensionality of the SERS data. The first and second principal component (PC) scores were utilized to construct two-dimensional plots and subsequently used as input variables for linear discriminant analysis (LDA) to develop a supervised classification model for differentiation of *Vibrio alginolyticus*, *Vibrio parahaemolyticus*, and *Vibrio vulnificus*. By constructing two-dimensional plots, the spectral clustering and differences among the three *Vibrio* species based on their spectral profiles was visualized. The LDA model was constructed using the selected PCA scores to maximize separation between bacterial groups. The classification performance of the PCA-LDA model was evaluated using leave-one-out cross-validation, and the classification accuracy was determined by comparing the predicted class assignments with the actual species identities.

### 2.5. PCA–LDA Model Validation Using Blinded Samples

The PCA–LDA model was first developed using 35 known bacterial isolates, which served as the reference dataset for generating discriminant functions that separate the three *Vibrio* strain groups. After the model was established, to evaluate the predictive performance of the model, eight additional bacterial isolates, cultured independently on different days using the same experimental protocol, were excluded from the training dataset and used as a blinded test set. The reference identities of these isolates were withheld during the prediction process. Each of these unknown samples was then classified into one of the three *Vibrio* species according to the highest probability score calculated by the LDA. Following prediction, the assigned classifications were compared with the known reference identities to assess the model’s predictive performance.

### 2.6. PLS-DA Model Development and Classification Performance

The SERS spectral profiles of the 35 known bacterial isolates used in the PCA-LDA model were subsequently analyzed using partial least squares-discriminant analysis (PLS-DA) to further evaluate the classification performance of *Vibrio alginolyticus*, *V. parahaemolyticus*, and *V. vulnificus* using OriginLab Pro version 2024 (OriginLab Corporation, Northampton, MA, USA). The model was developed by correlating spectral variables (X matrix) with categorical class information (Y matrix), and latent variables (LVs) were generated to maximize class discrimination. The score plots derived from the selected latent variables were used to visualize the separation among the three *Vibrio* species. Receiver-operating-characteristic (ROC) curves were used to further evaluate the model’s performance, while sensitivity and specificity were determined by calculating the area under the curve (AUC). The predicted class values obtained from the PLS-DA model were compared with the observed class values to evaluate model fitting. The calibration performance was computed using the coefficient of determination (R^2^) and root mean square error of calibration (RMSEC). OriginLab Pro version 2024 (OriginLab Corporation, Northampton, MA, USA) was used to create the calibration plots after RMSEC was initially calculated in Microsoft Excel 365 (Microsoft Corporation, Redmond, WA, USA).

## 3. Results and Discussion

Upon completion of bacterial preparation, SERS measurement, and spectral processing, the acquired Raman spectra were subjected to multivariate statistical analysis to evaluate the biochemical differentiation among the *Vibrio* strains. The following section presents the average SERS spectra and PCA results, with an emphasis on strain-specific Raman signatures across the fingerprint region. Pairwise comparisons and LDA were subsequently employed to assess the robustness and accuracy of the classification model in resolving closely related *Vibrio* strains based on their molecular fingerprints.

### 3.1. Average SERS Spectral Profiles of Vibrio Strains

**Figure 2:**
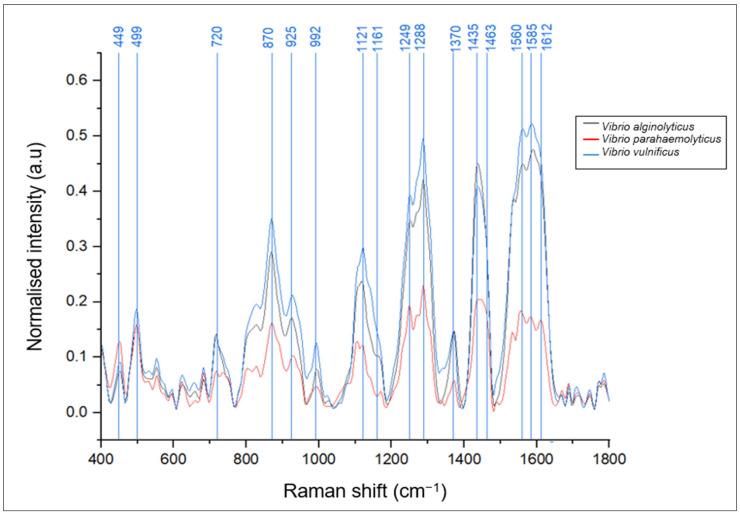
Average SERS spectra of bacteria *Vibrio* from *Vibrio alginolyticus*, *Vibrio parahaemolyticus*, and *Vibrio vulnificus*.

The average SERS spectra of *Vibrio alginolyticus*, *V. parahaemolyticus*, and *V. vulnificus* revealed distinct spectral fingerprints within the region of 400–1800 cm^−1^, as shown in Figure 2. The spectra were analyzed by baseline corrected and area normalization across the 400–1800 cm^−1^ region and displayed variations in Raman peak positions and bacteria intensities, indicating differences in the biochemical compositions of the three *Vibrio* species. Prominent Raman peaks were observed at 449, 499, 720, 870, 885, 925, 992, 1121, 1161, 1249, 1288, 1370, 1435, 1463, 1560, 1585, and 1612 cm^−1^ corresponding to molecular vibrations associated with proteins, nucleic acids, and lipids. Their common attributes are presented in Table 1. The Raman peak assignments were considered within ±10 cm^−1^ to account for slight peak variations caused by bacterial composition and SERS conditions. Among the species, *V. vulnificus* demonstrated noticeably higher intensities in the amide I (1600–1700 cm^−1^) and amide III (1200–1350 cm^−1^) regions, suggesting either a higher protein content or enhanced plasmonic interactions under the applied conditions [42,43]. In contrast, *V. parahaemolyticus* demonstrated lower peak intensities across multiple bands, potentially due to reduced SERS enhancement or lower concentration of Raman-active biomolecules. The average SERS spectra also revealed clear species-specific vibrational signatures among the tested *Vibrio* strains. *V. parahaemolyticus* exhibited a relatively higher intensity at 449 cm^−1^, corresponding to C–C skeletal vibrations and ring-deformation modes associated with polysaccharide and cell wall components [21]. The increased intensity likely reflects the variations in cell envelope composition, particularly in polysaccharide-associated structures. Previous studies have reported that *V. parahaemolyticus* produces extracellular polysaccharides that contribute to biofilm formation and surface-associated properties [44]. Variations in *Vibrio* bacterial surface components, such as cell wall structures and extracellular polymeric substances, can contribute to Raman spectral profiles and can influence species-specific discrimination among closely related bacteria [45]. In contrast, *V. alginolyticus* showed a distinct peak at 1435 cm^−1^, located within the CH_2_ bending region of fatty acids and lipids (1400–1450 cm^−1^), indicating differences in membrane lipid composition [46,47]. Previous studies have shown the presence of unique monounsaturated and polyunsaturated fatty acids in *V. alginolyticus* that are commonly detected in bacterial fatty acid profiles and can correlate with high CH_2_ deformation signals in Raman spectra [48]. Meanwhile, *V. vulnificus* displayed unique peaks at 1560, 1585, and 1612 cm^−1^, which fall within the aromatic C=C stretching region (1550–1620 cm^−1^), commonly linked to aromatic ring structures, nucleic acid bases, or protein amide components [49,50]. These distinctive spectral fingerprints emphasize the potential of SERS for rapid and specific identification of *Vibrio* species based on their molecular composition. The SERS spectra contain numerous bands, and assigning specific biomolecules is challenging due to the multiplexing of the wide variety of biomolecules in the mucus. To address this, PCA is applied to reduce the dimensionality of the SERS spectral data. PCA highlights the key features that contribute to the variance between groups, referred to as principal components (PCs). The loadings of the peaks along these PCs reflect their contribution to the variance observed among bacterial groups [51]. The loading plots were used to identify which specific vibrational bands contributed most significantly to intergroup separation, laying the foundation for downstream classification using PCA-LDA [52].

### 3.2. Principal Component Analysis (PCA) of Vibrio Species

**Figure 3:**
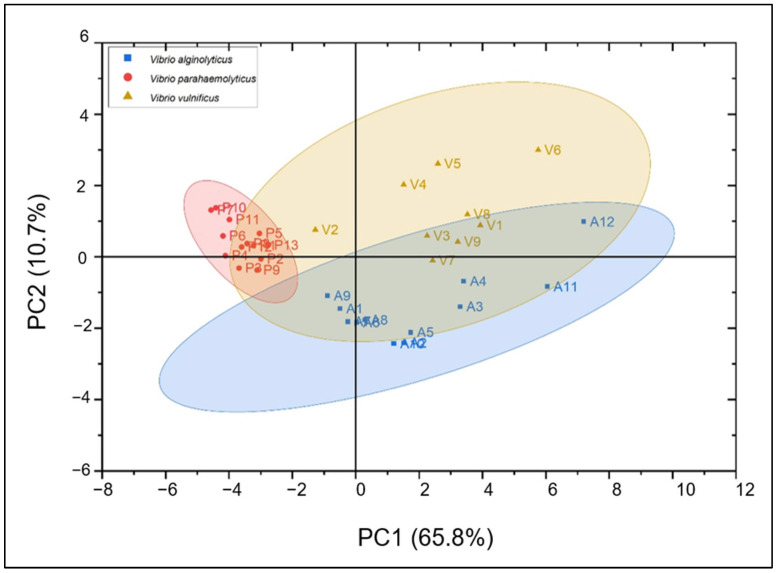
PCA score plot of Raman spectral profiles for three *Vibrio* strains. *Vibrio alginolyticus* (blue), *Vibrio parahaemolyticus* (red), and *Vibrio vulnificus* (yellow).

PCA was employed to reduce the dimensionality of the SERS spectral dataset, visualize the clustering patterns and to explore the intrinsic variance among the three *Vibrio* species. Overall, the calculated values of the first four principal components of each group were observed in Table S1. Figure 3 shows the PCA scatter plot of the SERS spectral data of all bacterial pellets with 95% confidence ellipse was observed, including *Vibrio alginolyticus*, *Vibrio parahaemolyticus*, and *Vibrio vulnificus*. The spectra of *V. alginolyticus* and *V. vulnificus* showed by black and green dots, respectively, are observed in the positive side of PC1 in PCA scatter plot, while *V. parahaemolyticus* shown by the red dots is clustered in the negative side of PC1. The PCA scatter plot showed the differentiation of 65.8% of data variability in PC1 and 10.7% in PC2, with a cumulative of 71.5% of data variance. The scatter plot demonstrated overlapping but distinguishable cluster regions of all three species. These clustering trends align with findings from previous studies, where PCA effectively distinguished bacterial species based on their unique Raman spectral fingerprints. Jarvis et al. (2006) and Chisanga et al. (2018) demonstrated that SERS, combined with PCA, was able to accurately identify and discriminate between closely related bacterial strains, attributing the variance to differences in molecular composition [32,53]. These studies support the efficacy of PCA in rapid bacterial identification and classification using label-free SERS analysis.

**Table 1:** Tentative peak assignment of significant peaks of Raman spectral profile of *Vibrio* bacteria.

Peak cm^−1^	Functional Group	Tentative Peak Assignment	Reference
449	C–C skeletal vibrations	Polysaccharide backbone (ring deformation)	[54]
499	S–S/C–S stretching	Amino acid sulfur side chain/cysteine	[55,56]
682	Ring breathing mode	Nucleic acid base ring	[57,58]
720	Ring breathing mode	Nucleic acid base ring (Adenine)	[59]
870	C-O-C skeletal mode/C–C skeletal vibrations	Carbohydrates ring structure/Polysaccharide structure	[60,61]
885	C-O-C skeletal mode	Carbohydrate components and cell wall polysaccharides	[60,61]
890	C-O-C skeletal mode	Carbohydrate components and cell wall polysaccharides	[60,61]
925	C-O-C skeletal mode/C–C skeletal vibrations	Carbohydrates ring structure/Polysaccharide structure	[60,61,62]
952	C–N stretching	Protein backbone	[47,54]
972	C–N stretching	Protein backbone	[50]
992	C–C stretching/C–N stretching	Phenylalanine ring breathing/Protein backbone	[50,63]
1121	C–O/C–O–C stretching vibrations	Carbohydrate components and polysaccharides	[61,64]
1142	C–C/C–N stretching	Protein backbone or ring modes of nucleic acid bases	[58]
1156	C–C/C–N stretching	Protein backbone or lipid-protein interface	[58,65]
1161	C–C/C–N stretching	Carotenoids or lipid-protein interface	[32,66]
1249	Amide III	Protein secondary structure (β-sheet)	[47]
1288	Amide III	Protein backbone (α-helix structure)	[54,57]
1312	CH_2_/CH_3_ deformation	Lipids (saturated fatty acids)	[50]
1336	Adenine ring breathing	Nucleic acids (DNA/RNA)	[50]
1370	CH_3_ deformation/COO^−^ stretching	Proteins, Fatty acid, or lipid carboxylate	[67,68]
1383	CH_3_ deformation/COO^−^ stretching	Fatty acid or lipid carboxylate	[47,50]
1435	CH_2_ bending	Fatty acid, lipids	[47,69]
1463	CH_2_ bending/C-H bending	Lipids, protein, or phospholipid content	[70]
1533	Amide II	N–H bending, C–N stretching in proteins	[49,50]
1542	Amide II	Protein	[49,50]
1560	C=C stretching/CH_2_ deformation	Aromatic amino acids or nucleic acid bases	[71,72]
1574	C=C stretching	Aromatic amino acids or nucleic acid bases	[71]
1585	C=C stretching	Aromatic amino acids or nucleic acid bases	[61]
1597	C=C stretching	Phenylalanine/Tryptophan (aromatic amino acids)	[73,74]
1612	C=C stretching	Aromatic amino acids or nucleic acid bases	[21]
1642	Amide I	Protein secondary structure (α-helix or β-sheet)	[47,69]

#### 3.2.1. PCA-Based Pairwise Species Discrimination

**Figure 4:**
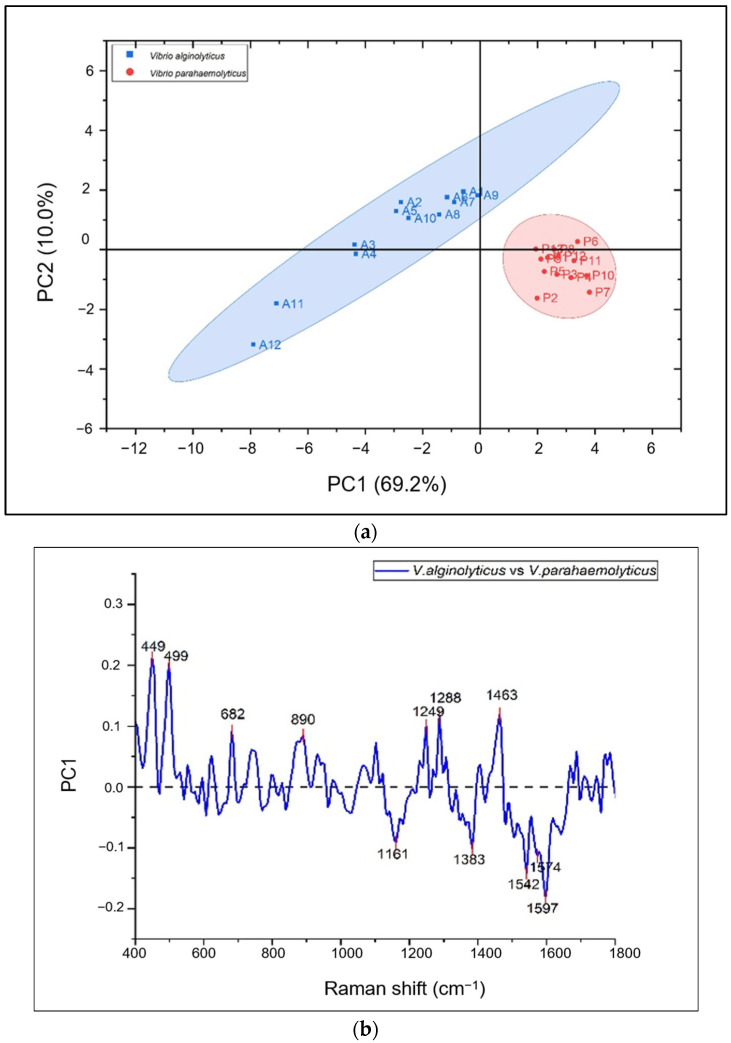
(**a**) PCA scatter plot of Raman spectral profile of *Vibrio alginolyticus* (blue) and *Vibrio parahaemolyticus* (red). (**b**) PCA Loadings of Raman spectra for *V. alginolyticus* and *V. parahaemolyticus* group. *V. parahaemolyticus* in the positive region, *V. alginolyticus* positioned in the negative region.

To further investigate the discriminative ability of SERS to distinguish bacterial cell pellets based on their unique biochemical profiles, pairwise PCA was employed to compare the spectral data sets of each bacterial group against one another. Figure 4a shows the PCA comparison between *V. alginolyticus* and *V. parahaemolyticus*. In the scatter plot, no overlapping region was observed, indicating a distinct cluster between the two species. The variance explained by PC1 and PC2 is 69.2% and 10.0%, respectively. The PCA loading plot illustrated in Figure 4b shows the clustering of *Vibrio parahaemolyticus* in the positive region of PC1 and *Vibrio alginolyticus* in the negative region. The loading peaks were selected using a 50% cutoff. The positive loadings associated with *V. parahaemolyticus* in PC1 include significant peaks at 449 cm^−1^ (C–C skeletal vibrations), 499 cm^−1^ (S–S/C–S stretching), 682 cm^−1^ (Ring breathing mode), 890 cm^−1^ (C-O-C skeletal mode), 1249 cm^−1^ (Amide III), 1288 cm^−1^ (Amide III), and 1463 cm^−1^ (CH_2_ bending or C-H bending). Meanwhile, the negative loadings related to *V. alginolyticus* include peaks at 1161 cm^−1^ (C–C/C–N stretching), 1383 cm^−1^ (CH_3_ deformation/COO^−^ stretching), 1542 cm^−1^ (amide II), 1574 cm^−1^ (aromatic C=C stretching), and 1597 cm^−1^ (C=C stretching). All peak tentative assignments are shown in Table 1. The assignment of peaks was conducted based on their characteristic functional groups, with an accepted deviation of ±10 cm^−1^ from the reported literature values to accommodate potential instrumental variations. In *V. parahaemolyticus*, the significance higher peak was observed in peaks associated with protein, lipid, and carbohydrate components compared with *V. alginolyticus*. The band at 499 cm^−1^ is associated with sulfur-containing amino acids and proteins, while the amide III bands at 1249 and 1288 cm^−1^ are related to protein structure [21,32]. The band at 890 cm^−1^ is assigned to carbohydrate components and may reflect differences in cell-surface polysaccharides [60,61]. Meanwhile, *V. alginolyticus* showed higher peaks that are mainly associated with proteins and lipids. The higher amide II peak may indicate differences in protein composition or membrane-associated proteins, while the stronger 1383 cm^−1^ peaks may be related to differences in amino acid residues and lipid functional groups [47,50]. The observed spectral profile differences are due to variations in their biochemical composition of bacteria cell envelopes, such as membrane proteins, surface polysaccharides, and lipid functional groups [32,49,75]. These variations allow a clear separation of *V. alginolyticus* and *V. parahaemolyticus* in the PCA model.

**Figure 5:**
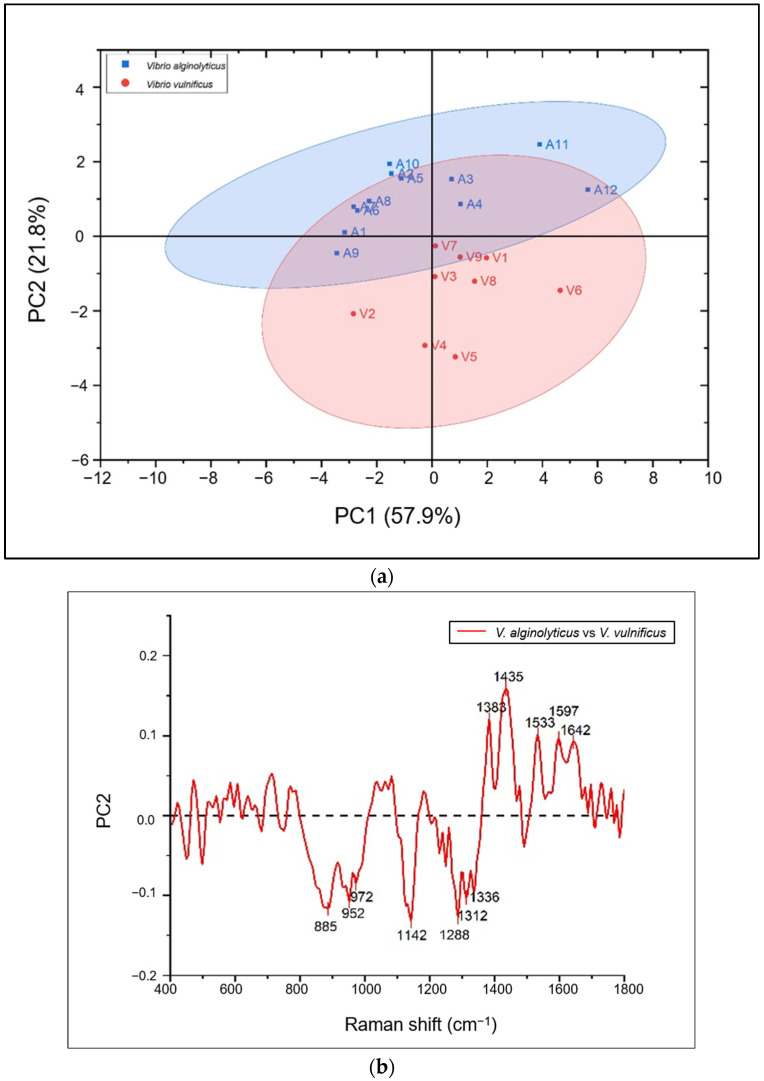
(**a**) PCA scatter plot of Raman spectral profile of *Vibrio alginolyticus* (blue) and *V. vulnificus* (red). (**b**) PCA Loadings of Raman spectra for *V. alginolyticus* and *V. vulnificus* group. *V. alginolyticus* in the positive region, *V. vulnificus* in the negative region.

Figure 5a presents the PCA comparison between *V. alginolyticus* and *V. vulnificus*. The PCA scatter plot shows a clear separation between the two species, with no overlapping data points, although there is an overlap in the 95% confidence ellipses. This indicates distinct clustering and separation of spectral profiles between the two *Vibrio* species, despite a partial overlap in the 95% confidence ellipses. PC1 captures 57.9% of the overall variance in the dataset, while PC2 accounts for an additional 21.8%. As shown in Figure 5b, the PCA loading plot highlights the spectral variables contributing to the distinction between the two species, with *V. alginolyticus* primarily located in the positive region of PC2 and *V. vulnificus* positioned in the negative region. Positive PC2 peaks that are significantly higher in *V. alginolyticus* included 1383 cm^−1^ (CH_3_ deformation/COO^−^ stretching), 1435 cm^−1^ (CH_2_ bending), 1533 cm^−1^ (amide II), 1597 cm^−1^ (C=C stretching), and 1642 cm^−1^ (amide I). In contrast, the *V. vulnificus* in negative PC2 has significantly higher peaks in 885 cm^−1^ ((C-O-C) skeletal mode), 952 cm^−1^ (C–N stretching 972 cm^−1^ (C-N stretching), 1142 cm^−1^ (C–C/C–N stretching) 1288 cm^−1^ (Amide III), 1312 cm^−1^ (CH_2_/CH_3_ deformation), and 1336 cm^−1^ (Adenine ring breathing). These spectral variations reflect key biochemical differences between the species and support the robustness of PCA for species discrimination using Raman spectroscopy.

**Figure 6:**
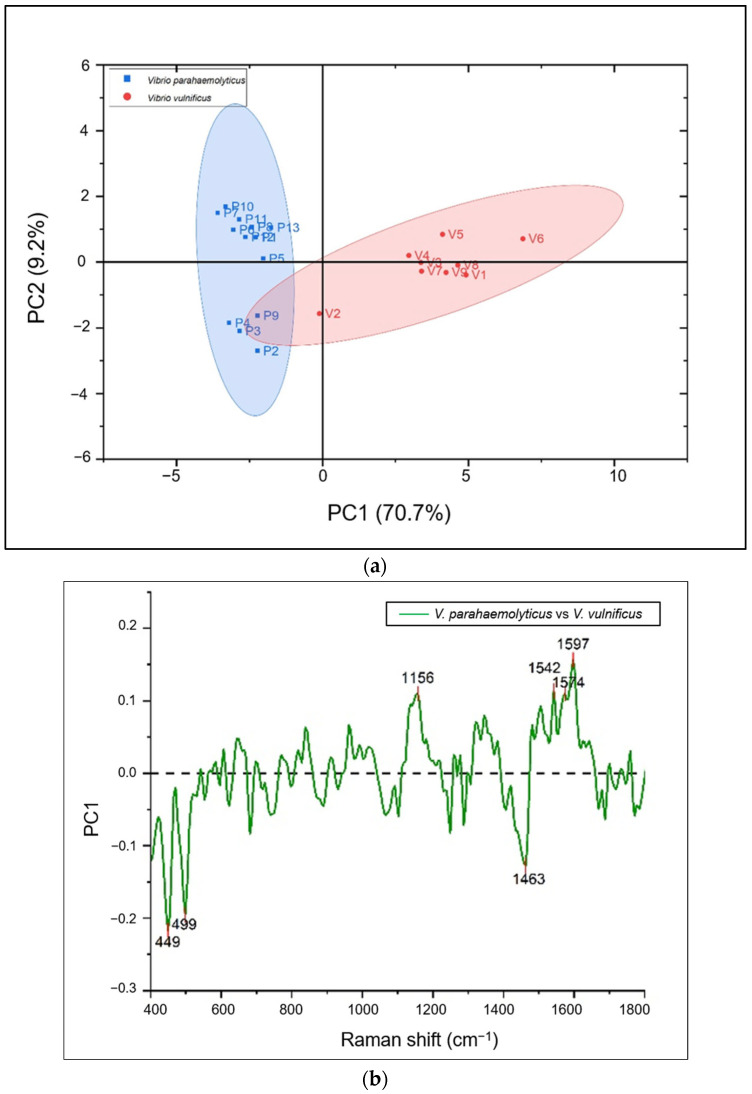
(**a**) PCA scatter plot of Raman spectral profile of *V. parahaemolyticus* (blue) and *V. vulnificus* (red). (**b**) PCA Loadings of Raman spectra for *V. parahaemolyticus* and *V. vulnificus* group. *V. vulnificus* in the positive region, *V. parahaemolyticus* in the negative region.

Figure 6a displays the PCA comparison between *V. parahaemolyticus* and *V. vulnificus*. The PCA scatter plot reveals a clear separation between the two species, with slight overlap in the confidence ellipses but no overlapping data points, indicating distinct clustering and well-separated spectral profiles. PC1 showed 70.7% of the variance, while PC2 represents 9.2%. The corresponding PCA loading plot in Figure 6b illustrates the variables contributing to this separation. *V. vulnificus* clusters predominantly in the positive region of PC1, whereas *V. parahaemolyticus* appears mostly in the negative region. Key loading peaks were selected based on a 50% cutoff. Positive PC1 peaks associated with *V. vulnificus* include 1156 cm^−1^ (C–C/C–N stretching), 1542 cm^−1^ (amide II), 1574 cm^−1^ (aromatic C=C stretching), and 1597 cm^−1^ (aromatic C=C stretching). In contrast, negative PC1 peaks linked to *V. parahaemolyticus* include 449 cm^−1^ (C–C skeletal vibrations), 499 cm^−1^ (S–S/C–S stretching), and 1463 cm^−1^ (CH_2_ bending/C-H bending). The aromatic C=C stretching (1574, 1597 cm^−1^) in *V. vulnificus* may be due to the presence of significantly higher nucleic acid or aromatic amino acid content, which is consistent with *V. vulnificus* and other known virulence factors that include nucleic acid-binding proteins and iron acquisition systems [76,77].

Based on the findings above, the three *Vibrio* species exhibited common Raman spectral features, including amide I and II bands (∼1640–1550 cm^−1^), amide III bands (∼1200–1300 cm^−1^), lipid-associated C–H bending vibrations (∼1450 cm^−1^), and phosphate-related nucleic acid signals (∼1080 cm^−1^), consistent with fundamental biochemical components shared among bacteria, as reported in previous SERS studies [78,79]. However, the variation in the relative intensities of all these shared peaks allowed for effective species discrimination. Particularly *V. parahaemolyticus* showed significantly stronger signals at ∼499 cm^−1^, associated with C–S stretching, suggesting a distinct sulfur metabolism possibly linked to environmental adaptability and virulence [49]. Meanwhile, *V. vulnificus* exhibited enhanced amide III and II signals, pointing to a higher protein or peptidoglycan content in its cell envelope [80]. Such spectral differences likely stem from variations in the outer membrane of each species, particularly differences in outer membrane proteins (OMPs) and lipopolysaccharide structures, which have been shown to influence Raman signal [23,36]. Additionally, higher significant signals from aromatic amino acids (∼1000–1030 cm^−1^) were observed in *V. vulnificus*, potentially reflecting species-specific enrichment of lipoproteins and related metabolic byproducts [23]. In contrast, *V. alginolyticus* and *V. parahaemolyticus* exhibited lower intensities at some of these positions, in line with known differences in membrane protein compositions. Overall, these results highlight the sensitivity of SERS combined with multivariate analysis in capturing subtle biochemical distinctions, enabling reliable species-level identification of closely related *Vibrio* pathogens [36,78,81].

#### 3.2.2. PCA-LDA Classification and Discrimination of *Vibrio* Species

PCA-LDA is an excellent discrimination model, which was employed for the differentiation of the SERS spectra of the *Vibrio* strain bacterial pellets. Table 2 summarizes the results of Linear Discriminant Analysis (LDA) classification and leave-one-out cross-validation using the first four principal components. Each of the three *Vibrio* species *V. alginolyticus*, *V. parahaemolyticus*, and *V. vulnificus* was correctly identified through LDA, achieving a classification accuracy of 100% for all groups with no misclassifications observed. During cross-validation, the model maintained a high level of classification accuracy, with only one sample misclassified. The cross-validation process, which involved sequentially removing one data point from the dataset to validate the model built from the remaining data, also resulted in 97.06% accuracy across all species. This shows that the LDA model has high discriminatory capability to distinguish between the spectral profiles of these closely related *Vibrio* strains under the evaluated experimental conditions. These results were supported by Jarvis et al. (2006) and Cheng et al. (2021), who reported that SERS coupled with multivariate analysis enables accurate bacterial identification based on subtle biochemical differences [32,82]. The single misclassified sample during cross validation is likely associated with inherent spectral variability within bacterial groups, which has been commonly observed in SERS bacterial classification studies, where minor classification errors may occur despite high predictive accuracy due to biological variation among bacterial cells [79]. Detailed classification probabilities and cross-validation results from the LDA model are provided in Table S2, where misclassified samples are indicated by an asterisk (*) and their assigned groups are highlighted.

**Figure 7:**
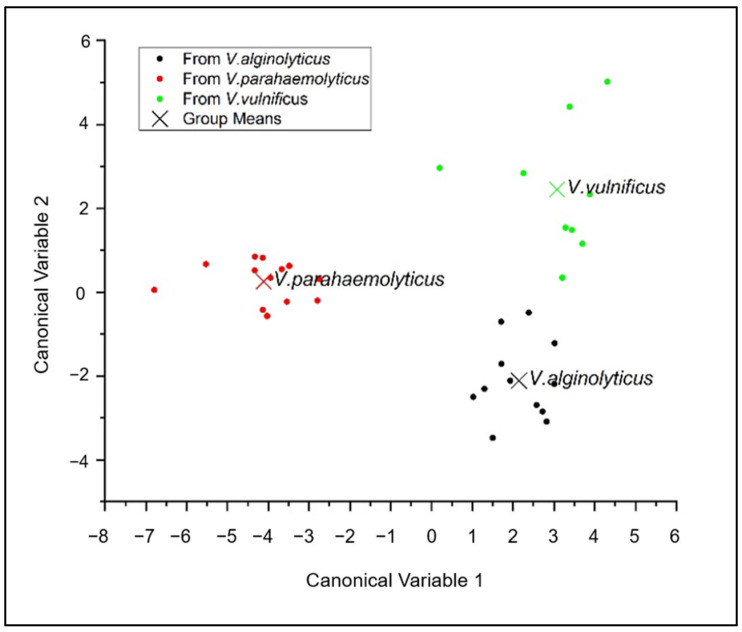
Two-dimensional scatter plot of the first two canonical variables from LDA of *Vibrio alginolyticus*, *Vibrio parahaemolyticus*, and *Vibrio vulnificus*. Group means are indicated with a cross.

Figure 7 presents the LDA scatter plot, which clearly illustrates the separation among the three *Vibrio* species. Each group clusters distinctly along the canonical variables, with *V. alginolyticus*, *V. parahaemolyticus*, and *V. vulnificus* occupying separate regions of the plot. The group means, represented by “X” markers, further emphasizes this separation, indicating strong class discrimination. The PCA-LDA model achieved 100% classification accuracy and 97.06% cross-validation accuracy, with no overlap observed in the classification data. However, one *V. alginolyticus* data point was misclassified as *V. vulnificus* during cross-validation. This minor misclassification slightly reduces the cross-validation accuracy but still reinforces the robustness of LDA in differentiating the Raman spectral profiles. This finding aligns with previous PCA analyzes, which also demonstrated distinct grouping of bacterial species and supports earlier reports on the successful application of PCA-LDA and other supervised machine-learning models for species-level bacterial detection using Raman or SERS spectra [69]. The clear segregation supports the presence of species-specific biochemical signatures, likely influenced by differences in membrane composition, such as outer membrane proteins and lipopolysaccharide structures, which contribute to variations in the Raman spectra [49,83]. Compared to Raman analysis using PCA only, the LDA-supervised framework markedly boosts classification performance by leveraging class labels during the sample training [84].

#### 3.2.3. Prediction of Blinded Samples by the PCA–LDA Model

The PCA-LDA model was created using 35 known references of *Vibrio* bacteria sample and was then constructed into a discriminant function by separating the three *Vibrio* species, *V. alginolyticus*, *V. parahaemolyticus*, and *V. vulnificus*. To evaluate the predictive capability of the PCA-LDA model, eight additional bacterial samples as blinded samples were analyzed, where their reference identities were withheld during the analysis. The summary of unknown sample classified is presented in Table 3. All eight blinded samples were successfully assigned to one of the three *Vibrio* species in the PCA–LDA model, with no isolates remaining unclassified or yielding ambiguous predictions. Out of the eight isolates, three were classified into *V. alginolyticus*, three as *V. parahaemolyticus*, and the remaining two as *V. vulnificus*. The corresponding percentages were 37.5%, 37.5%, and 25.0%, respectively. As shown in Table 4, all eight isolates were correctly predicted according to their reference identities. The confusion matrix showed no false-positive or false-negative classifications among the three species, yielding a prediction accuracy of 100%. These results demonstrate the potential of the PCA–LDA model for species-level discrimination of *Vibrio* isolates based on their Raman spectral fingerprints. However, further validation with larger and more diverse isolated collections is required to confirm its broader applicability. This validated that current PCA-LDA models are reliable for discriminating closely related *Vibrio* species. A previous study reported that a Raman spectroscopy study combined with PCA-LDA was able to achieve over 90% sensitivity and specificity for differentiating clinical isolated by their gram type with 93.6% accuracy [85].

### 3.3. PLS-DA Modeling and Validation of Vibrio Species

**Figure 8:**
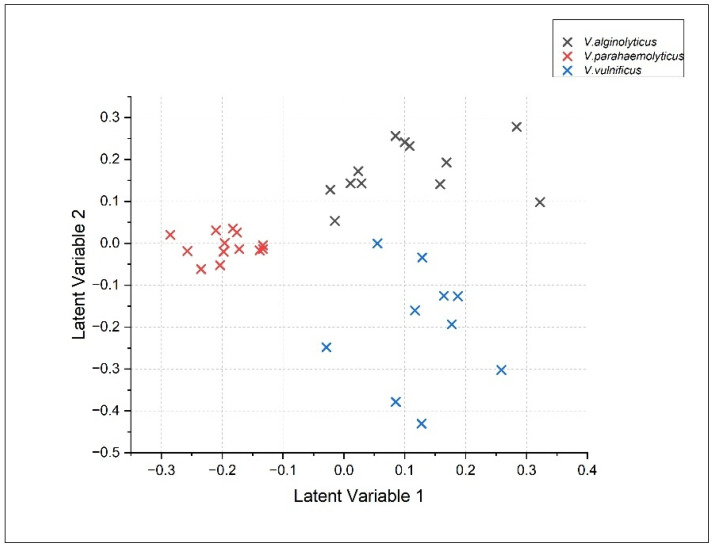
PLS-DA score plot showing species-level separation of *Vibrio alginolyticus*, *V. parahaemolyticus*, and *V. vulnificus*.

**Figure 9:**
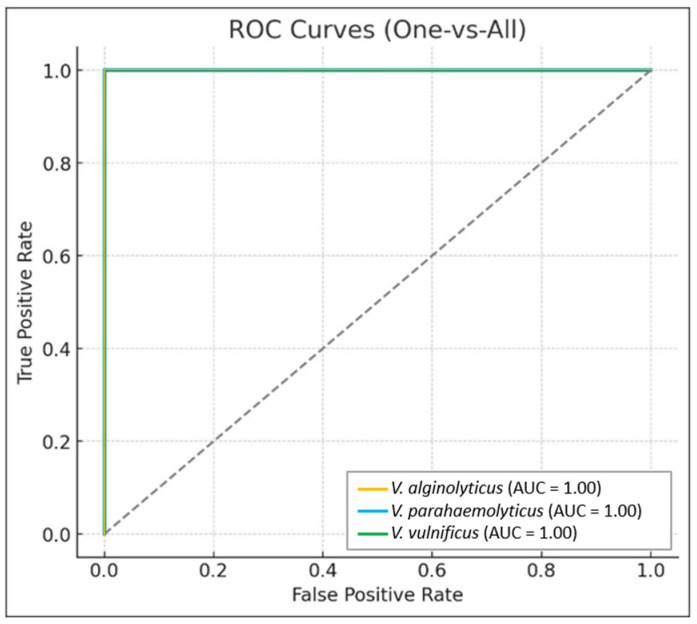
ROC Curves Showing Species-Specific Diagnostic Performance. The overlapping curves for the three *Vibrio* species are due to identical (AUC = 1.00). The curves represent *V. alginolyticus* (yellow line), *V. parahaemolyticus* (blue line), and *V. vulnificus* (green line).

In PLS-DA modelling, the PLS-DA score plot (Figure 8) showed a clear separation among *Vibrio alginolyticus*, *V. parahaemolyticus*, and *V. vulnificus*, with each species forming distinct clusters along the first two latent variables. This indicates that the created model successfully captured species-specific spectral signatures. *V. parahaemolyticus* formed a tightly grouped cluster, reflecting low intra-species variability, while *V. alginolyticus* and *V. vulnificus* showed slightly broader dispersion, suggesting greater biological heterogeneity within these species. These results showed similarity with the PCA in Figure 3. This separation was further supported by ROC curve analysis (Figure 9), where all species achieved an AUC value of 1.00, confirming perfect sensitivity and specificity of the model within the evaluated dataset. Such clear classification performance is similar with previous research that used chemometric modeling and vibrational spectroscopy to identify bacteria, where PLS-DA and ROC analyze showed high discriminatory capability for differentiating closely related bacteria [86]. However, the AUC value obtained from this model dataset reflects the performance of the current *Vibrio* model and may vary when evaluated using a larger and more diverse sample, including *Vibrio* isolates from natural environment, fish mucus, or tissue sample. Supplementary Table S3 presents the PLS latent variable scores, while Supplementary Table S4 provides the ROC coordinate values for *Vibrio* species classification, further supporting model performance.

**Figure 10:**
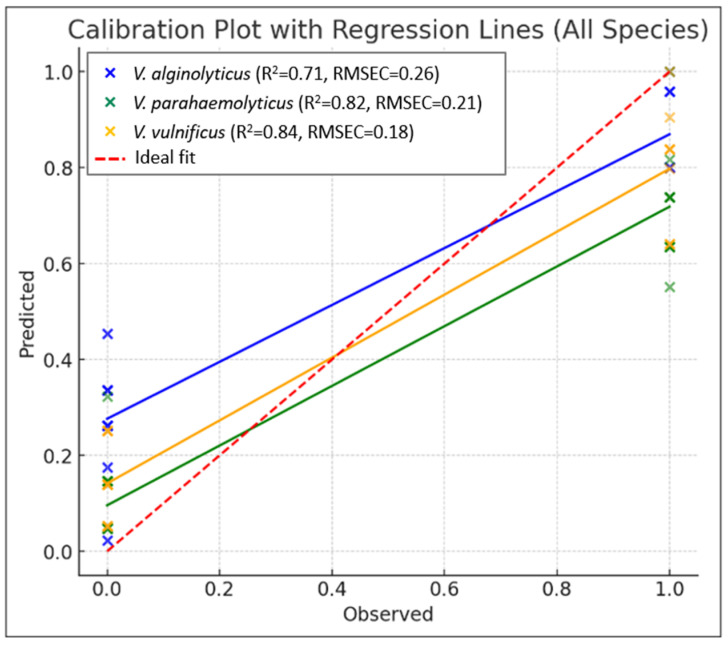
Calibration plots of predicted versus observed values for *V. alginolyticus, V. parahaemolyticus*, and *V. vulnificus* using PLS-DA. The regression lines are shown for *V. alginolyticus* (blue), *V. parahaemolyticus* (green), and *V. vulnificus* (orange), with the red dashed line representing the ideal fit.

The calibration plots (Figure 10) were then performed, the predictive accuracy of the model, with *V. vulnificus* showing the strongest performance (R^2^ = 0.84, RMSEC = 0.183), followed by *V. parahaemolyticus* (R^2^ = 0.82, RMSEC = 0.206), while *V. alginolyticus* demonstrated weaker calibration (R^2^ = 0.71, RMSEC = 0.256), indicating greater prediction variability. The calibration plot data for the PLS-DA prediction models of *Vibrio* species classification are presented in Supplementary Table S5. The previous study reported that closely related species displaying greater biological heterogeneity frequently produced less stable calibration models when Raman spectroscopy and chemometric analysis were combined [87], and similar results have been reported in micro-Raman-based PLS-DA models, where PLS-DA showed classification accuracy of 73% while SVM showed 89% performance. The study states that the greater heterogeneity presents difficulties for model reliability and ascribed this decreased calibration stability to the spectral variability among bacterial species [88]. The relatively weaker calibration for *V. alginolyticus* may reflect intrinsic variability in its metabolic or phenotypic profiles, which has been noted as a challenge in modeling generalist bacterial species. The predictive performance of *V. vulnificus* and *V. parahaemolyticus*, which exhibit R^2^ values exceeding 0.80, is consistent with the standards of robust microbial classification models in spectroscopy-based studies [89]. These calibration results represent the current model performance under a controlled experiment condition. Further evaluation using independent datasets such as natural environment collected *Vibrio* isolates and aquaculture related samples, with increased biological replicates required to assess model transferability.

Taken together, the latent variable separation (Figure 8), perfect classification by ROC curves (Figure 9), and species-dependent calibration performance (Figure 10) highlight the robustness of PLS-DA for *Vibrio* discrimination and classification of *Vibrio* species. SERS coupled with PLS-DA accurately classified *Vibrio* species, underscoring its potential for monitoring aquaculture pathogens. However, the previous study has highlighted the necessity of assessing model performance using larger and more diverse datasets to ensure generalizability across biological systems, meaning that the reliability of such models heavily depends on robust validation [90]. Furthermore, it has been demonstrated that adding variable importance to projection (VIP) analysis improves model interpretability by locating the most discriminant spectral regions; VIP scores greater than 1.0 are trustworthy indicators of class separation [91]. With these improvements, vibrational spectroscopy may become a useful tool for routine surveillance of *Vibrio* pathogens in aquaculture by facilitating the creation of straightforward and rapid diagnostic models.

## 4. Conclusions

This study demonstrated that surface-enhanced Raman spectroscopy (SERS) combined well with multivariate chemometric approaches for the rapid differentiate of three closely related *Vibrio* species (*V. alginolyticus*, *V. parahaemolyticus*, and *V. vulnificus*). PCA-LDA analysis revealed that the first two principal components (PC1 = 65.8% and PC2 = 10.7%) effectively captured most of the spectral variability, with 100% classification accuracy achieved through LDA within the reference dataset, highlighting distinct biochemical differences among the species, particularly in protein, lipid, and amino acid-associated spectral features. Complementing this, the PLS-DA model further demonstrated strong discriminatory ability, with clear species-specific clustering, perfect classification performance as evidenced by ROC curves (AUC = 1.00), and strong predictive calibration for *V. parahaemolyticus* and *V. vulnificus* (R^2^ > 0.80), although *V. alginolyticus* exhibited weaker calibration due to greater intra-species heterogeneity. Together, these findings confirm that both unsupervised–supervised (PCA-LDA) and supervised (PLS-DA) approaches successfully captured the molecular variations that are associated with the species level to distinguish these pathogenic species. Importantly, integrating SERS with multivariate analysis provides a promising label-free platform for *Vibrio* detection and classification, offering a reliable tool for pathogen monitoring in aquaculture and underscoring the value of outer membrane composition in shaping spectral signatures. The findings support the potential of SERS, combined with multivariate analysis, as a rapid, label-free method, and reliable tool for species-level identification and classification of *Vibrio* pathogens, emphasizing the importance of outer membrane composition in shaping the Raman spectral signatures of these pathogenic bacteria. The present study represents a controlled proof-of-concept using pure bacterial cultures prepared under laboratory standardized conditions. Further validation is required using larger isolated collections of mixed bacterial cultures and other aquaculture samples such as water, fish mucus, and tissue homogenates. In addition, future studies should evaluate the analytical sensitivity of the method, including the detection limit and classification performance across different bacterial concentrations, to establish its suitability for routine pathogen detection and further monitoring in aquaculture.

## Figures and Tables

**Table 2:** LDA classification and cross-validation based on the first four PCs.

Reference	Classification				Cross-Validation			
	*Vibrio alginolyticus*	*Vibrio parahemolyticus*	*Vibrio vulnificus*	Total	*Vibrio alginolyticus*	*Vibrio parahemolyticus*	*Vibrio vulnificus*	Total
*Vibrio alginolyticus*	12	0	0	12	11	0	1	12
	100.00%	0.00%	0.00%	100.00%	91.67%	0.00%	8.33%	100.00%
*Vibrio parahemolyticus*	0	13	0	13	0	13	0	13
	0.00%	100.00%	0.00%	100.00%	0.00%	100.00%	0.00%	100.00%
*Vibrio vulnificus*	0	0	9	9	0	0	9	9
	0.00%	0.00%	100.00%	100.00%	0.00%	0.00%	100.00%	100.00%
Total	12	13	9	35	12	13	9	35
	35.29%	38.24%	26.47%	100.00%	32.35%	38.24%	29.41%	100.00%

**Table 3:** Classification of eight unknown bacterial samples into *Vibrio* species using PCA–LDA.

	*V. alginolyticus*	*V. parahaemolyticus*	*V. vulnificus*	Total
Count	3	3	2	8
Percent	37.50%	37.50%	25.00%	100.00%

**Table 4:** Confusion matrix of PCA–LDA classification for eight blinded *Vibrio* isolates.

Actual Identity (Reference)	Predicted: *V. alginolyticus*	Predicted: *V. parahaemolyticus*	Predicted: *V. vulnificus*	Total
*V. alginolyticus*	3	0	0	3
*V. parahaemolyticus*	0	3	0	3
*V. vulnificus*	0	0	2	2
Total	3	3	2	8

## Data Availability

Data will be made available on request from the corresponding author.

## Supplementary Materials

The following supporting information can be downloaded at: https://www.mdpi.com/article/10.3390/biology15151300/s1, Table S1. Values of the first four principal components of each group; Figure S1. Growth morphology of three *Vibrio* strains on TCBS agar plates: (a) *Vibrio alginolyticus*, (b) *Vibrio parahaemolyticus*, and (c) *Vibrio vulnificus*; Table S2. Probabilities of the LDA classification and cross-validation, with misclassified data points marked with an asterisk (*) and assigned groups highlighted; Table S3. Partial Least Squares (PLS) latent variable scores; Table S4. ROC curve coordinates for *Vibrio* species classification models; Table S5. Calibration plot data for PLS-DA prediction models of *Vibrio* species classification.

## Abbreviations

The following abbreviations are used in this manuscript:

SERS	surface-enhanced Raman spectroscopy
PCA-LDA	principal component analysis with linear discriminant analysis
PLS-DA	partial least squares discriminant analysis
TCBS	thiosulphate citrate bile salts sucrose
PCR	Polymerase Chain Reaction
RNA	Ribonucleic Acid
DNA	deoxyribonucleic acid
NP	Nanoparticles
PCA	principal component analysis
LDA	Linear discriminant analysis
ROC	Receiver Operating Characteristic
TSB	tryptic soy broth
OD	Optical density
AUC	area under the curve
RMSEC	root means square error of calibration
