# Supplementary material for: Discrimination of Closely Related Vibrio Strains by Label-Free Surface-Enhanced Raman Spectroscopy (SERS)
Source: Biology (Basel). 2026 Aug 5;15(15):1300. doi: 10.3390/biology15151300 (PMC13465455; doi:10.3390/biology15151300)
Supplement: Supplementary file 1 [file biology-15-01300-s001.zip › biology-4435935-supplementary.pdf]

Supplementary material

The following supporting information included, Table S1. Values of the first four principal components of each group; Figure S1. Growth morphology of three *Vibrio* strains on TCBS agar plates: (a*) Vibrio alginolyticus*, (b) *Vibrio parahaemolyticus*, and (c) *Vibrio vulnificus*; Table S2. Probabilities of the LDA classification and cross-validation, with misclassified data points marked with an asterisk (*) and assigned groups highlighted; Table S3. Partial Least Squares (PLS) latent variable scores; Table S4. ROC curve coordinates for *Vibrio* species classification models; Table S5. Calibration plot data for PLS-DA prediction models of *Vibri*o species classification.

Table S1. Values of the first four principal components of each group.

Group	PC1	PC2	PC3	PC4
V. alginolyticus	-0.49081	-1.45718	1.58339	-0.18326
V. alginolyticus	1.5159	-2.41909	0.40693	0.03368
V. alginolyticus	3.30312	-1.4002	-0.23897	-0.25617
V. alginolyticus	3.4119	-0.68756	-0.02051	-0.2906
V. alginolyticus	1.74273	-2.12207	0.37012	-0.11564
V. alginolyticus	0.03832	-1.84475	0.93571	0.11425
V. alginolyticus	-0.23968	-1.82024	0.67427	-0.2625
V. alginolyticus	0.29086	-1.77316	0.30378	-0.02681
V. alginolyticus	-0.88461	-1.09263	1.54644	0.37111
V. alginolyticus	1.20815	-2.43399	-0.41555	-0.13796
V. alginolyticus	6.05236	-0.83916	-1.90282	-1.11955
V. alginolyticus	7.1978	0.98307	-2.19422	-1.26123
V. parahaemolyticus	-3.22681	0.315	0.00578	-1.06655
V. parahaemolyticus	-2.9893	-0.06027	-2.72779	1.32199
V. parahaemolyticus	-3.68112	-0.31667	-1.84628	1.25745
V. parahaemolyticus	-4.11035	0.03367	-1.72091	1.26467
V. parahaemolyticus	-3.03867	0.6561	-0.40429	-0.25875
V. parahaemolyticus	-4.18823	0.58548	0.80969	-0.40752
V. parahaemolyticus	-4.56621	1.31056	-0.5091	-1.68054
V. parahaemolyticus	-3.42867	0.37078	0.50487	-0.91754
V. parahaemolyticus	-3.11163	-0.37152	-1.10288	1.1086
V. parahaemolyticus	-4.41301	1.37193	0.21896	-1.31023
V. parahaemolyticus	-3.98548	1.04497	0.53548	-0.76326
V. parahaemolyticus	-3.60031	0.27679	0.022	-0.86117
V. parahaemolyticus	-2.76859	0.32254	0.56682	-0.83778
V. vulnificus	3.93321	0.88308	-0.03617	0.36351
V. vulnificus	-1.27144	0.76061	-0.38809	2.14381
V. vulnificus	2.25345	0.58812	0.88821	0.57654
V. vulnificus	1.51598	2.02342	1.33109	1.47683
V. vulnificus	2.59547	2.61075	1.79377	1.17262
V. vulnificus	5.75836	2.99224	-0.59074	-1.00958
V. vulnificus	2.42833	-0.10139	0.5972	0.36709
V. vulnificus	3.52635	1.18863	0.53683	0.67503
V. vulnificus	3.22262	0.4221	0.46701	0.51947

Table S2. Probabilities of the LDA classification and cross-validation. The misclassified data point is marked with an asterisk (*) and the group it is assigned is marked in yellow.

Classification				Cross-validation			
**References**	**Probability**			**References**	**Probability**		
	V.alginolyticus	*V.parahaemolyticus*	*V.vulnificus*		*V.alginolyticus*	*V.parahaemolyticus*	*V.vulnificus*
V.alginolyticus	0.99994	7.76674E-9	5.99465E-5	*V.alginolyticus*	0.99988	2.02301E-8	1.15507E-4
V.alginolyticus	1	2.82862E-13	3.11955E-7	*V.alginolyticus*	1	2.48701E-13	3.54676E-7
V.alginolyticus	0.99998	7.70723E-13	2.3151E-5	*V.alginolyticus*	0.99996	9.92576E-13	3.57819E-5
V.alginolyticus	0.99807	7.33857E-12	0.00193	*V.alginolyticus*	0.99753	1.31472E-11	0.00247
V.alginolyticus	1	9.74206E-13	8.53692E-7	*V.alginolyticus*	1	1.29192E-12	1.14663E-6
V.alginolyticus	0.99999	7.99532E-10	1.19332E-5	*V.alginolyticus*	0.99998	1.79697E-9	1.88503E-5
V.alginolyticus	1	9.28566E-8	8.67353E-7	*V.alginolyticus*	1	1.51637E-7	1.15658E-6
V.alginolyticus	1	2.58734E-8	2.68398E-6	*V.alginolyticus*	1	4.01183E-8	3.97463E-6
V.alginolyticus	0.99416	8.65511E-8	0.00584	*V.alginolyticus*	0.98414	3.40203E-7	0.01586
V.alginolyticus	1	4.68706E-10	1.55009E-8	*V.alginolyticus*	1	1.30787E-9	7.61714E-9
V.alginolyticus	1	3.54735E-12	1.51603E-6	*V.alginolyticus*	1	4.11335E-12	2.3217E-6
V.alginolyticus	0.97068	2.13071E-9	0.02932	*V.alginolyticus**	0.07482	1.11872E-8	0.92518
V.parahaemolyticus	2.10995E-9	1	1.09359E-13	*V.parahaemolyticus*	4.63648E-9	1	2.94018E-13
V.parahaemolyticus	2.38329E-11	1	1.35323E-13	*V.parahaemolyticus*	5.53441E-11	1	5.40985E-13
V.parahaemolyticus	4.16406E-10	1	1.50774E-12	*V.parahaemolyticus*	1.45259E-9	1	6.70366E-12
V.parahaemolyticus	1.13748E-11	1	2.91144E-13	*V.parahaemolyticus*	2.10416E-11	1	1.0552E-12
V.parahaemolyticus	1.42039E-9	1	1.69955E-11	*V.parahaemolyticus*	2.66226E-9	1	3.10654E-11
V.parahaemolyticus	3.82302E-9	1	7.6503E-11	*V.parahaemolyticus*	8.54322E-9	1	1.57407E-10
V.parahaemolyticus	1.53111E-17	1	1.02743E-21	*V.parahaemolyticus*	1.08118E-22	1	8.87797E-29
V.parahaemolyticus	1.96268E-8	1	7.60677E-12	*V.parahaemolyticus*	3.59096E-8	1	2.02697E-11

V.parahaemolyticus	8.02584E-7	1	7.61825E-9	*V.parahaemolyticus*	2.05606E-6	1	1.67915E-8
V.parahaemolyticus	9.6805E-15	1	3.47994E-17	*V.parahaemolyticus*	1.27442E-15	1	7.30705E-18
V.parahaemolyticus	3.96356E-11	1	1.08896E-12	*V.parahaemolyticus*	8.65704E-11	1	3.09564E-12
V.parahaemolyticus	7.88451E-10	1	7.41923E-14	*V.parahaemolyticus*	1.69561E-9	1	1.91239E-13
V.parahaemolyticus	2.05702E-6	1	1.78322E-9	*V.parahaemolyticus*	2.30052E-6	1	3.01328E-9
V.vulnificus	0.00151	4.6136E-13	0.99849	*V.vulnificus*	0.00207	1.36788E-12	0.99793
V.vulnificus	3.66193E-5	2.38497E-4	0.99972	*V.vulnificus*	9.75747E-4	0.0761	0.92293
V.vulnificus	0.00138	1.2829E-12	0.99862	*V.vulnificus*	0.00174	3.67111E-12	0.99826
V.vulnificus	2.46149E-9	1.10213E-15	1	*V.vulnificus*	2.56221E-10	7.7263E-16	1
V.vulnificus	6.71757E-11	3.75994E-19	1	*V.vulnificus*	2.7144E-14	3.87023E-23	1
V.vulnificus	9.73413E-6	1.19153E-10	0.99999	*V.vulnificus*	3.42422E-5	2.52939E-9	0.99997
V.vulnificus	0.25404	2.29561E-11	0.74596	*V.vulnificus*	0.39359	1.24724E-10	0.60641
V.vulnificus	2.10203E-5	3.08824E-15	0.99998	*V.vulnificus*	3.26275E-5	5.06773E-15	0.99997
V.vulnificus	0.00546	1.49252E-13	0.99454	*V.vulnificus*	0.00771	4.35801E-13	0.99229

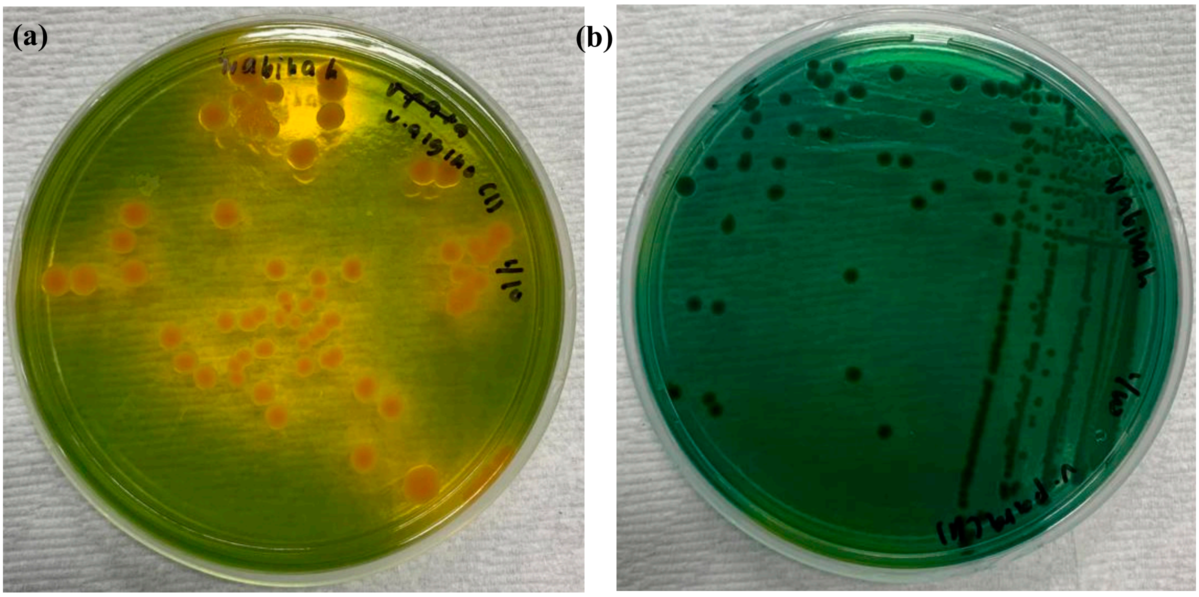

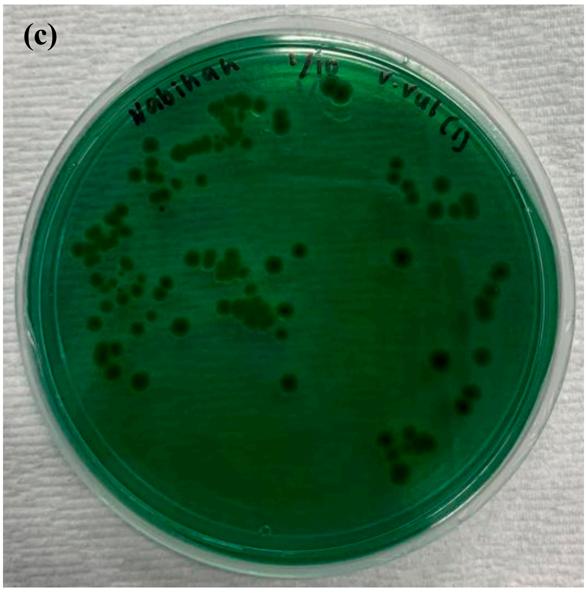

Figure S1. Growth morphology of three *Vibrio* strains on TCBS agar plates: (a) *V. alginolyticus,* (b) *V. parahaemolyticus,* and (c) *V. vulnificus*.

Table S3. Partial Least Squares (PLS) Latent Variable Scores

Sample id	Group	LV1	LV2
S1	*V.alginolyticus*	-0.02239	0.12795
S2	*V.alginolyticus*	0.09973	0.24178
S3	*V.alginolyticus*	0.16855	0.19312
S4	*V.alginolyticus*	0.15803	0.14116
S5	*V.alginolyticus*	0.10787	0.23213
S6	*V.alginolyticus*	0.02869	0.14304
S7	*V.alginolyticus*	0.01091	0.14307
S8	*V.alginolyticus*	0.02333	0.17233
S9	*V.alginolyticus*	-0.01518	0.05381
S10	*V.alginolyticus*	0.08462	0.2563
S13	*V.parahaemolyticus*	-0.2103	0.03125
S14	*V.parahaemolyticus*	-0.13301	-0.01326
S15	*V.parahaemolyticus*	-0.17578	0.0263
S16	*V.parahaemolyticus*	-0.19755	-0.01999
S17	*V.parahaemolyticus*	-0.17227	-0.01341
S18	*V.parahaemolyticus*	-0.20352	-0.05202
S19	*V.parahaemolyticus*	-0.28522	0.0205
S20	*V.parahaemolyticus*	-0.18259	0.03569
S21	*V.parahaemolyticus*	-0.13311	-0.00492
S22	*V.parahaemolyticus*	-0.25724	-0.01835
S23	*V.parahaemolyticus*	-0.23464	-0.06141
S24	*V.parahaemolyticus*	-0.19584	0.00131
S25	*V.parahaemolyticus*	-0.13867	-0.01665
S26	*V.vulnificus*	0.18701	-0.12626
S27	*V.vulnificus*	-0.02896	-0.24773
S28	*V.vulnificus*	0.11667	-0.15994

S29	*V.vulnificus*	0.08523	-0.37814
S30	*V.vulnificus*	0.12735	-0.43041
S31	*V.vulnificus*	0.25843	-0.30199
S32	*V.vulnificus*	0.12854	-0.03373
S33	*V.vulnificus*	0.17688	-0.19305
S34	*V.vulnificus*	0.1641	-0.12517
S11	*V.alginolyticus*	0.28358	0.27848
S12	*V.alginolyticus*	0.32194	0.09831
S35	*V.vulnificus*	0.05482	-1.04382E-4

Table S4. ROC Curve Coordinates for *Vibrio* Species Classification Models

Species	Cut-off	False Positive Rate (1-Specificity)	True Positive Rate (Sensitivity)
V. alginolyticus	-1.37334	1	1
V. alginolyticus	-0.33944	0.95652	1
V. alginolyticus	-0.22713	0.91304	1
V. alginolyticus	-0.1172	0.86957	1
V. alginolyticus	-0.07071	0.82609	1
V. alginolyticus	-0.04095	0.78261	1
V. alginolyticus	-0.01871	0.73913	1
V. alginolyticus	-0.00726	0.69565	1
V. alginolyticus	0.00936	0.65217	1
V. alginolyticus	0.02579	0.6087	1
V. alginolyticus	0.0326	0.56522	1
V. alginolyticus	0.04165	0.52174	1
V. alginolyticus	0.05103	0.47826	1
V. alginolyticus	0.06618	0.43478	1
V. alginolyticus	0.08244	0.3913	1
V. alginolyticus	0.08937	0.34783	1
V. alginolyticus	0.09387	0.30435	1
V. alginolyticus	0.09595	0.26087	1
V. alginolyticus	0.12158	0.21739	1
V. alginolyticus	0.15521	0.17391	1
V. alginolyticus	0.17553	0.13043	1
V. alginolyticus	0.22851	0.08696	1
V. alginolyticus	0.28947	0.04348	1
V. alginolyticus	0.56972	0	1
V. alginolyticus	0.82984	0	0.91667
V. alginolyticus	0.8396	0	0.83333
V. alginolyticus	0.86674	0	0.75
V. alginolyticus	0.88524	0	0.66667
V. alginolyticus	0.8901	0	0.58333
V. alginolyticus	0.90869	0	0.5
V. alginolyticus	0.95539	0	0.41667
V. alginolyticus	0.99857	0	0.33333

V. alginolyticus	1.02559	0	0.25
V. alginolyticus	1.05611	0	0.16667
V. alginolyticus	1.07969	0	0.08333
V. alginolyticus	2.08855	0	0
V. parahaemolyticus	-1.11243	1	1
V. parahaemolyticus	-0.09464	0.95455	1
V. parahaemolyticus	-0.07564	0.90909	1
V. parahaemolyticus	-0.07322	0.86364	1
V. parahaemolyticus	-0.061	0.81818	1
V. parahaemolyticus	-0.04434	0.77273	1
V. parahaemolyticus	-0.03766	0.72727	1
V. parahaemolyticus	-0.03655	0.68182	1
V. parahaemolyticus	-0.03565	0.63636	1
V. parahaemolyticus	-0.03034	0.59091	1
V. parahaemolyticus	-0.01984	0.54545	1
V. parahaemolyticus	-0.00309	0.5	1
V. parahaemolyticus	0.01042	0.45455	1
V. parahaemolyticus	0.0248	0.40909	1
V. parahaemolyticus	0.03893	0.36364	1
V. parahaemolyticus	0.05555	0.31818	1
V. parahaemolyticus	0.08645	0.27273	1
V. parahaemolyticus	0.10953	0.22727	1
V. parahaemolyticus	0.12685	0.18182	1
V. parahaemolyticus	0.1511	0.13636	1
V. parahaemolyticus	0.16566	0.09091	1
V. parahaemolyticus	0.26568	0.04545	1
V. parahaemolyticus	0.53371	0	1
V. parahaemolyticus	0.74746	0	0.92308
V. parahaemolyticus	0.7983	0	0.84615
V. parahaemolyticus	0.80865	0	0.76923
V. parahaemolyticus	0.85417	0	0.69231
V. parahaemolyticus	0.91193	0	0.61538
V. parahaemolyticus	0.93882	0	0.53846
V. parahaemolyticus	0.95567	0	0.46154
V. parahaemolyticus	0.96516	0	0.38462
V. parahaemolyticus	0.9974	0	0.30769
V. parahaemolyticus	1.03676	0	0.23077
V. parahaemolyticus	1.07115	0	0.15385
V. parahaemolyticus	1.2308	0	0.07692
V. parahaemolyticus	2.36652	0	0
V. vulnificus	-0.16614	0.96	1
V. vulnificus	-0.14443	0.92	1
V. vulnificus	-0.10563	0.88	1
V. vulnificus	-0.07729	0.84	1

V. vulnificus	-0.06227	0.8	1
V. vulnificus	-0.04599	0.76	1
V. vulnificus	-0.03269	0.72	1
V. vulnificus	-0.01676	0.68	1
V. vulnificus	-0.00725	0.64	1
V. vulnificus	-0.00264	0.6	1
V. vulnificus	0.00147	0.56	1
V. vulnificus	0.00505	0.52	1
V. vulnificus	0.01785	0.48	1
V. vulnificus	0.0318	0.44	1
V. vulnificus	0.03494	0.4	1
V. vulnificus	0.0368	0.36	1
V. vulnificus	0.05055	0.32	1
V. vulnificus	0.06615	0.28	1
V. vulnificus	0.10002	0.24	1
V. vulnificus	0.1464	0.2	1
V. vulnificus	0.1635	0.16	1
V. vulnificus	0.17619	0.12	1
V. vulnificus	0.19435	0.08	1
V. vulnificus	0.21015	0.04	1
V. vulnificus	0.45911	0	1
V. vulnificus	0.7129	0	0.9
V. vulnificus	0.73201	0	0.8
V. vulnificus	0.83911	0	0.7
V. vulnificus	0.94899	0	0.6
V. vulnificus	0.97341	0	0.5
V. vulnificus	0.99309	0	0.4
V. vulnificus	1.01939	0	0.3
V. vulnificus	1.06653	0	0.2
V. vulnificus	1.15683	0	0.1
V. vulnificus	2.22317	0	0

Table S5. Calibration plot data for PLS-DA prediction models of *Vibrio* species classification.

Species	Probability Bin	Mean Predicted Probability	Mean Observed Probability	Sample Count
V. alginolyticus	0.0	0.035437	0	18
V. alginolyticus	0.1	0.166057	0	3
V. alginolyticus	0.2	0.269250	0	1
V. alginolyticus	0.3	0.309690	0	1
V. alginolyticus	0.8	0.862223	1	6
V. alginolyticus	0.9	0.955385	1	2
V. alginolyticus	1.0	1.000000	1	4
V. parahaemolyticus	0.0	0.010522	0	16
V. parahaemolyticus	0.1	0.137648	0	5
V. parahaemolyticus	0.3	0.364380	0	1

V. parahaemolyticus	0.7	0.747460	1	2
V. parahaemolyticus	0.8	0.837683	1	3
V. parahaemolyticus	0.9	0.951990	1	4
V. parahaemolyticus	1.0	1.000000	1	4
V. vulnificus	0.0	0.014715	0	19
V. vulnificus	0.1	0.161295	0	4
V. vulnificus	0.2	0.210150	0	2
V. vulnificus	0.6	0.698840	1	1
V. vulnificus	0.7	0.732010	1	2
V. vulnificus	0.9	0.971038	1	4
V. vulnificus	1.0	1.000000	1	3
